# An In vitro Evaluation of the Effect of Four Dentin Bonding System on the Bond Strength between Quartz Fiber Post and Composite Core

**Published:** 2014-03

**Authors:** M. Shirinzad, Sh. Ebadi, M. Shokripour, MA. Darabi

**Affiliations:** a Dept. of Operative Dentistry, School of Dentistry, Hamedan University of Medical Science, Hamedan, Iran; b Dept. of Operative Dentistry, School of Dentistry, Ghazvin University of Medical Science, Ghazvin, Iran; c Dept. of Operative Dentistry, School of Dentistry, Hamedan University of Medical Science, Hamedan, Iran; d Dentist

**Keywords:** Bond strength, Composite core, Dentin-bonding agents, Fiber post

## Abstract

**Statement of Problem:** A strong bond of fiber post to resin core, as well as to dentin would critically ensure the durability of restorations in endodontically treated teeth.

**Purpose:** The purpose of this study was to evaluate the effect of etch-and-rinse dentin bonding systems on the bond strength between resin core and fiber post after application of 24% hydrogen peroxide.

**Materials and Method: **24 fiber posts (RTD; St. Egèven, France) were treated with 24% hydrogen peroxide for 10 minutes. They were randomly divided into 4 groups (n=6) based on the bonding agent used: Group P: Prime&Bond, Group O: One Step, Group S: Single Bond and Group E: Excite. Each group was prepared according to the manufacturer’s instructions. For all posts, a flowable composite core (ÆliteFlo; Bisco, USA) was built-up over the bonded area. Each specimen was sectioned to produce 2 sticks, 1mm in thickness and underwent microtensile bond strength (µTBS). Data were analyzed using one-way ANOVA at the 0.05 level. The fractured surfaces of all sticks were evaluated by stereomicroscope (× 20). Scanning electron microscopy(SEM) assessment of two sticks from each group was performed to evaluate the surface morphology.

**Results:** The means and SDs of µTBS were: Group P: 10.95±1.74; Group S: 10.25±2.39; Group E: 9.52±2.07; and Group O: 9.12±1.34. There was no statistically significant difference in bond strength means between the groups tested (p> 0.05).

**Conclusion:** The results of this study indicated the bonding agents used had no significant influence on the bond strength of fiber post to composite core.

## Introduction

In recent years, use of fiber posts due to aesthetics, bonding to dentin and resistance to corrosion [1-2] has been increased in the restoration of endodontically treated teeth [3-7].

An ideal post provides good retention of the core and supports the core to prevent loosening of the cemented crown [8]. Long-term service of a post/core restoration is related to the type of the post, core buildup material and the quality of the adhesion between core and post [9-10].

Improvement of the adherence between the resinous core and fiber post is one of the important factors for durability of final restoration. It has been reported that there is not any chemical interaction between composite resin and the epoxy resin matrix of fiber posts [11-12]. Surface treatment of fiber posts is a common technique to provide chemical and/or mechanical interaction between the post and surrounding composite. Chemical techniques such as etching with hydrofluoric acid, application of potassium permanganate, silane and hydrogen peroxide was suggested to enhance the surface roughness [13-17]. It has been reported that etching with hydrogen peroxide provided an easy, effective, conservative and stronger adhesion between post surface and resin composite in comparison with other corrosive liquids [14, 18-20].

It is suggested to use an intermediate adhesive layer on the FRC post before core build up for promoting micromechanical and chemical bonding by increasing surface wetting and providing oxygen inhibited layer of non-reacted monomers [21-23]. Due to elastic characteristics of this layer, it may be effective to release the polymerization stress of the core material, but the effect of type and the compositions of the bonding system on adhesion between fiber post and core are unclear [24]. Thus, this study was carried out to compare the effect of different bonding systems on bond strength of the resinous core and fiber post. The null hypothesis was that the bond strength between fiber post and composite core with the various bonding systems are not significantly different.

## Materials and Method

Twenty four white posts (#2, RTD; St. Egèven, France) were selected. The posts were made of unidirectional pretended quartz fiber, embedded in an epoxy resin matrix [4].

**Figure 1:**
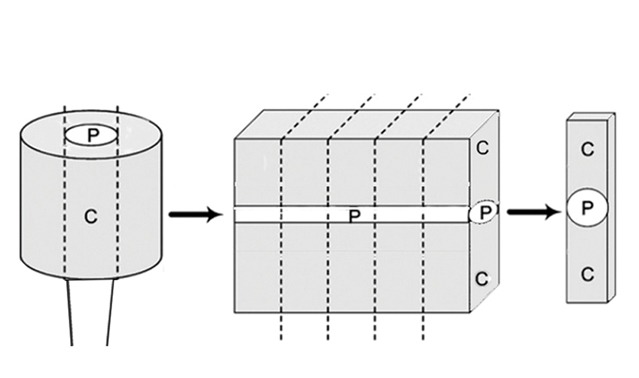
Showing schematic design of the specimen (p = Post and C = Core).

The posts were first immersed in a 24% hydrogen peroxide solution for 10 min at room temperature and were, then, rinsed with running water for 2 min and gently air-dried. The bonding agents, used in this study, were four light-curing etch-and-rinse bonding agent systems. The hydrogen-peroxide-treated posts were divided into four groups based on the bonding agent applied: Group P: Prime&Bond, Group O: One Step, Group S: Single Bond and Group E: Excite. Their compositions and instructions of application are summarized in Table 1. Each group was prepared according to the manufacturer’s instructions. Core build up was, then, performed by using a flowable composite (Ælite Flo, Bisco, USA). To build up the cores, each post was positioned upright on a glass slab, and fixed with a drop of sticky wax. A cylindrical matrix was, then, placed around the posts and adjusted so that the posts were exactly in the center. The matrix was 10 mm in diameter and the length was equal to the non-tapered portion of the post (6 mm). The flowable composite was applied to the post in 1-mm-thick increments and light cured, separately, using a halogen light curing unit (Degulux II; Degussa AG, Gschttsbereich Dental, D-63457 Hanau-Wolfgang, Germany) with an intensity of 450mW/cm [2]. Once the matrix was completely filled, the composite cylinder was removed from the glass slab. An additional 40s irradiation was performed from the bottom of the cylinder prior to the removal of the matrix to ensure optimal polymerization of the core material. Then, each cylinder was secured on the sample-holder of a thin sectioning machine (Hamco Inc.; Rochester, NY). 1-mm-thick sections were serially cut, perpendicular to the long axis of the post by using a water-cooled blade, and then two longitudinal cuts were performed. Finally, sticks with uniform thickness were prepared in which the posts were placed in the center and the build-up cores on each side (12 sticks for each group). Figure 1 shows the schematic design of each specimen.

**Table 1:** Dentin bonding systems studied and their composition

**Groups**	**Adhesive System**	**Coats** **Recommended**	**Manufacturer**	**Composition of Adhesive**
1 (P)	Prime & bond NT	1	Dentsply	PENTA, UDMA + T-resin (cross- linking agent) + D-resin (small hydrophilic molecule) , butylated hydroxitoluene , 4 – ethyl dimethyl aminobaenzoate, cetilamine hydrofluoride, acetone, silice nanofiller
2 (O)	One- Step	2	Bisco, Inc.	Bis- GMA, BPDM, HEMA, acetone
3 (S)	Single Bond	2	3M ESPE	Bis- GMA, HEMA, dimethacrylates, polyalkenoic acid copolymer, initiator, water, ethanol
4 (E)	Excite	1	Ivoclar Vivadent	Phosphonate monomer, HEMA, cross- linking agents, fumed silica, 25% ethanol

Every stick was glued with mad wolf to the two free-sliding components of a jig, which was mounted on the microtensile test machine (Micro Tensile Tester; T-61010K, Bisco, USA).

This set-up was conceived to apply tensile forces to the two opposite post-core interface. The specimens were loaded at a crosshead speed of 0.5 mm/min until failure occurred at any of the two stressed interfaces. Bond strength was expressed in mega Pascal (MPa), by dividing the load at failure (Newton) to the bonding surface area (mm^2^). The failure modes of samples were evaluated using a stereomicroscope (M6C-10, Russia) at × 20. The failure mode was evaluated as cohesive failure (failure in the post or core material), adhesive failure (failure at the interface of post and core) and mixed failure (adhesive- cohesive failure).

The evaluation of the fractured surfaces was performed using scanning electron microscopy (SEM) (Tescan; ZEGA II, SMU, Czech Republic) on two randomly selected fractured sticks of each group. Data were analyzed using one-way ANOVA test at the 0.05 significance level. 

## Results

The means and standard deviation values of microtensile bond strength test (µTBS) were as follows: group P (10.95±1.74), group S (10.25±2.39), group E (9.52± 2.07) and group O (9.12±1.34), respectively. The results indicated that there was no statistically significant difference between mean values of the studied groups (p> 0.05) (Table 2).

**Table 2:** Microtensile bond strength of fiber post to composite core

**Groups**	**Number**	**µTBS (MPa)** **(mean** **±SD)**	**F**	P.Value*
P	12	10.95±1.74	1.75	0.17
O	12	9.12±1.34		
S	12	10.25±2.39		
E	12	9.52±2.07		

*One-way ANOVA

**Figure 2:**
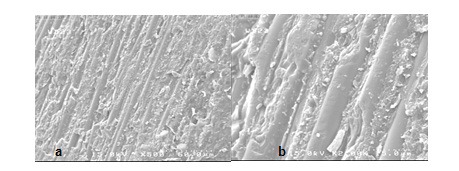
SEM views of the fractured surface showing adhesive failure in group P. a (×500) view is magnified in b (×2,000).

**Figure 3:**
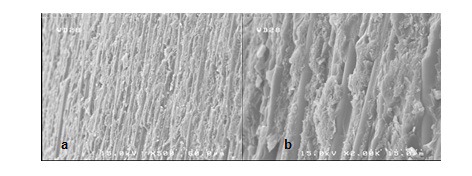
SEM views of the fractured surface showing adhesive failure in group O. a (×500) view is magnified in b (×2,000).

**Figure 4:**
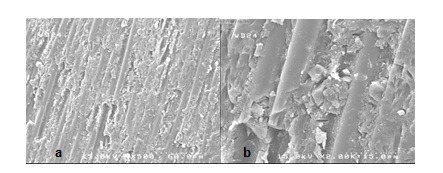
SEM views of the fractured surface showing adhesive failure in group S. a (×500) view is magnified in b (×2,000).

**Figure 5:**
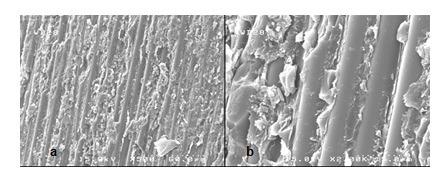
SEM views of the fractured surface showing adhesive failure in group E. a (×500) view is magnified in b (×2,000).

The stereomicroscopic evaluation revealed that all of the failures were occurring on the interface of post and core material (adhesive mode).

SEM assessment showed adhesive failure. The micrographs represented the exposed quartz fibers after treatment with hydrogen peroxide (Figures 2, 3, 4 and 5).

## Discussion

The long-term clinical maintenance of final restorations depends on the adhesion between the posts and composite cores [25-26] and increasing of bond strength of the posts to cores is necessary for a successful restoration.

The bond strength between the fiber post and core composite is a critical problem because the polymer matrix between the fibers is too much cross-linked and inactive; an issue that can lead to a weak adhesion between the post and the composite core material [27]. This study was also designed to evaluate the microtensile bond strength of quartz fiber post binding to composite by using the etch-and-rinse dentin bonding agents.

Hydrogen peroxide (H2O2) treatment is especially effective in enhancing bond strength of flowable composites to fiber posts due to the ability of these composites to penetrate into post surface irregularities which form semi-interpenetrating (semi-IPN) polymer matrix [28]. Studies reported increased bond strength of composite to FRC posts with a semi-IPN polymer matrix [29-30].The surface of fiber posts in this study was rinsed with hydrogen peroxide. Application of hydrogen peroxide, on the surface of FRC posts, is considered as a reliable conservative and easy technique for improving the bond strength between the posts and cores [18]. The researchers reported that surface treatment of quartz and glass fiber posts with hydrogen peroxide significantly enhance the bond strength due to its ability to remove the surface layer of the epoxy resin matrix of post and creating a better mechanical bonding between resin and fiber post [14, 18-20, 31]. It seems that the embedded free radicals of oxygen from the applied hydrogen peroxide may extrude in, depending on the dentin bonding type, especially the type of their solvent. Thus, two bonding agents containing acetone (Prime &Bond NT and One step) and other two bonding agents containing ethanol solvent (Single Bond and Excite) were chosen in this study.

Because of the low viscosity, the flowable composites are able to penetrate optimally within the post surface irregularities, taking the greatest advantage of the increase in surface area available for bonding following post surface pre-treatment [19]. Therefore, a flowable composite was selected as the core material in this study.

The method utilized for bond strength testing was the microtensile bond test that was performed according to previous studies, reported to be reliable for the evaluation of interfacial bond strength [3, 19]. Microtensile test allows the use of relatively small flat surface of specimens for a more uniform distribution of the loading stress, which limits the chance of cohesive failures, thus allowing for an appropriate assessment of interfacial bond strength [32-33]. Nonetheless, the push-out and pull-out tests are probably influenced by flows and non-uniform bonding in a manner similar to coronal bonding [34].

However, the results indicated there was no significant difference between the groups studied, in spite of their different solvent component. Studies reported that application of different bonding agents had no effect on bond strength of composite core to post which was in agreement with this study results [24, 35] but could influence the microtensile bond strength FRC post to dentin [36].

The stereomicroscope and SEM evaluation show ed that all of the failures were at the interface of post and core material, so the weakest bond strength was in the adhesion area of two materials.

This result was seen in the study by Balbosh et al. [37] and Wrbas et al. [38]. They stated the high failure rate of adhesive failure was a sign of the absence of bonding between fiber post and composite core material.

One of the limitations of the present study is that only one type of composite resin core and fiber post was evaluated. Future studies, using various cores and posts, are recommended. Another limitation is that pre-treatment of the post was immediately followed by the application of the bonding agent and composite core material. For simulation of oral conditions, it is better to perform other studies in clinical situations.

## Conclusion

Under the limitations of this in vitro study, the results revealed the etch-and-rinse bonding agent had no influence on the bond strength of the quartz fiber post to the composite core.
